# Aging is Associated with Prolonged Hospitalisation Stay in Pyogenic Liver Abscess—A 1:1 Propensity Score Matched Study in Elderly Versus Non-Elderly Patients

**DOI:** 10.21315/mjms2022.29.5.7

**Published:** 2022-10-28

**Authors:** Kai Siang Chan, Sameer P Junnarkar, Jee Keem Low, Cheong Wei Terence Huey, Vishal G Shelat

**Affiliations:** 1 Department of General Surgery, Tan Tock Seng Hospital, Singapore; 2 Lee Kong Chian School of Medicine, Nanyang Technological University, Singapore

**Keywords:** epyogenic liver abscess, elderly, multi-modal care, gas-forming, aging

## Abstract

**Background:**

Mortality of pyogenic liver abscess (PLA) is high ranging 10%–40%. Old age predicts outcomes in many diseases but there is paucity of data on PLA outcomes. We aim to compare the morbidity and mortality between elderly and non-elderly in PLA.

**Methods:**

This is a retrospective study from 2007–2011 comparing elderly (≥ 65 years old) and non-elderly (< 65 years old) with PLA. A 1:1 propensity score matching (PSM) was performed. Baseline clinical profile and outcomes were compared.

**Results:**

There were 213 patients (elderly patients = 90 [42.3%], non-elderly patients = 123 [57.7%]). Overall median age is 62 (interquartile range [IQR] = 53–74) years old. PSM resulted in 102 patients (51 per arm). Length of hospitalisation stay (LOS) was significantly longer in elderly patients in both unmatched (16 [IQR = 10–24.5] versus 11 [IQR = 8–19] days; *P* < 0.001) and matched cohorts (17 [IQR = 13–27] versus 11 [IQR = 7–19] days; *P* = 0.001). In-hospital mortality was significantly higher in elderly patients in the unmatched cohort (elderly patients = 21.1%, non-elderly patients = 7.3%; *P* = 0.003) but was insignificant following PSM (elderly patients = 15.7%, non-elderly patients = 9.8%; *P* = 0.219). Duration of antibiotic therapy and need for percutaneous drainage (PD) were comparable before and after PSM.

**Conclusion:**

Age ≥ 65 years old is associated with longer LOS. In-hospital mortality though higher in elderly patients, was not statistically significant.

## Introduction

Pyogenic liver abscess (PLA) remains the most common type of liver abscess, accounting for 48% of all visceral abscesses and 13% intra-abdominal abscesses (1). However, the incidence of PLA varies globally, ranging from 1.1 per 100,000 in Europe to 17.6 per 100,000 in Asia (2). Presentation of PLA remains non-specific, with fever, lethargy, malaise, right upper quadrant pain and jaundice (3). Therefore, a high clinical index of suspicion is required to conduct imaging studies for prompt PLA diagnosis. This allows for early intervention, which has been shown to improve outcomes in PLA (4–7).

Literature has established various risk factors and biomarkers which may be used to prognosticate PLA. Comorbidities such as diabetes mellitus and hypertension predict failure of percutaneous therapy (aspiration or drainage) and prolonged length of hospitalisation stay (LOS) (8). Imaging biomarkers such as the presence of multiple abscesses, large size or gas formation are poor prognostic factors for PLA (9–11). Old age is a risk factor that has been established to predict worse outcomes in many diseases, including hepatopancreatobiliary diseases (12–16). The Tokyo Guidelines 2018 (TG18) use age ≥ 75 years old as one of the criteria for moderate cholangitis, while the Ranson’s score and the modified Glasgow score use age > 55 years old as one of the components in severity stratification for acute pancreatitis (14–16).

Old age is associated with a reduction in vital capacity and lean body mass, reduced cardiac output and sarcopenia (17). In PLA, a retrospective study by Chen et al. (18) on 339 patients (age ≥ 65 years old: *n* = 118, age < 65 years old: *n* = 221) demonstrated that age ≥ 65 years old is associated with longer LOS with comparable mortality. However, another study by Law and Li (19) on 319 patients (age ≥ 65 years old [52.7%]) showed that age ≥ 65 years old is associated with a higher mortality rate. Furthermore, old age is associated with the confounding effect of co-morbidity, which may worsen outcomes (20). However, there is a paucity of literature on the real impact of age on outcomes in PLA. This study aims to address the confounding effect of comorbidities and clinical profile of patients and evaluate the real impact of age on outcomes in PLA using propensity score matching (PSM). We aim to compare the morbidity and mortality between the elderly (≥ 65 years old) and non-elderly (< 65 years old).

## Methods

This is a single-centre retrospective case-control study of patients with PLA from 2007 to 2011 at our university-affiliated tertiary hospital. Exclusion criteria were patients with amoebic liver abscess or tuberculosis liver abscess, infected liver cyst or hydatid cyst and patients aged < 18 years old. Traditionally, the elderly is defined as age of 65 years old or older (21). However, in more recent studies, a cutoff of 75 years old was more commonly used to define ‘elderly’ (22, 23). The TG18 (14) for acute cholangitis also included age > 75 years old as part of the criteria for moderate cholangitis. The reason for proposing a higher cut-off age for ‘elderly’ is because of the phenomenon of ‘rejuvenation’, where there is a delay of deterioration of physical function such as gait speed and grip strength by 5 years–10 years, compared with 10 years–20 years ago (21, 24). Nevertheless, we defined ‘elderly’ as aged 65 years old or older in our study as our study population included patients dating back to 2007 and a more balanced sample size if a cut-off of 65 years old was used (≥ 65 years old versus < 65 years old: 90 patients versus 123 patients; > 75 years old versus ≤ 75 years old: 43 patients versus 170 patients). For the purpose of this study, we will be referring to age ≥ 65 years old as elderly and age < 65 years old as non-elderly. This study’s conduct is in accordance with the STrengthening the Reporting of OBservational studies in Epidemiology (STROBE) statement for retrospective case-control studies (25). Deidentified data were pre-collected and the study team made no further patient contact for data collection purposes. No attempts were made by the study team to access the patients’ electronic medical records.

### Study Variables and Outcomes

Study variables include age, gender, American Society of Anesthesiologists (ASA) score, comorbidities, clinical presentation, biochemistry and radiological investigations. Radiological findings included number of abscesses, size of the largest abscess and presence of gas formation. Multiple abscesses were defined as the presence of more than one abscess. While there is no standardised definition for ‘large’ or ‘giant’ PLA, we defined them as > 4 cm–< 10 cm and ≥ 10 cm, respectively, following previous reports on PLA (5, 6). Study outcomes include LOS, duration of parenteral antibiotics, duration of the total course of antibiotics (including parenteral and oral), need for PD or surgical drainage, 30-day re-admission and in-hospital mortality. The 30-day re-admission was defined as readmission for PLA or associated condition within 30 days from the initial admission date. In-hospital mortality refers to incidence of mortality during the index hospitalisation stay.

### Treatment Protocol

A definitive diagnosis of PLA was made using computed tomography (CT) scan in all patients. Initial management of suspected PLA or any hepatopancreatobiliary infection was managed according to the Surviving Sepsis Campaign Guidelines for Management of Severe Sepsis and Septic Shock 2012 (26). Management of patients presenting with fever or septic shock included biochemistry investigations with at least one set of blood cultures before administration of parenteral broad-spectrum antibiotics within an hour of presentation as per our local antibiogram: a stat dose of amoxicillin-clavulanic acid 1.2 g and gentamicin at 5 mg/kg body weight. Gentamicin was omitted in patients with acute kidney injury. Patients with penicillin allergy were given a combination of cefazolin, metronidazole and gentamicin or third-generation cephalosporin (ceftriaxone) and metronidazole.

The overall management of PLA is summarised by our ‘liver abscess care bundle’ consisting of a multidisciplinary team of surgeons, infectious disease physicians, interventional radiologists and nurses (5). Subsequent choice of antibiotics was guided by culture sensitivity results (blood or pus cultures) and the local antibiotic stewardship programme. Solitary PLA < 4 cm was initially managed conservatively using antibiotic therapy. Percutaneous drainage (PD) was performed in the presence of ≥ 1 of any of the following indications: i) size of PLA > 4 cm; ii) presence of haemodynamic instability or need for vasopressor or inotropic support; iii) presence of gas formation or iv) failure of conservative management (defined as no improvement or worsening of clinical status or biochemistry markers after 3–5 days). PD was performed by interventional radiologists through radiological guidance (ultrasound or CT guided) with a 10–12 French pigtail catheter placement. Percutaneous aspiration was not performed in our institution in view of lower success rates (27).

Repeat radiological imaging (ultrasonography or CT) was performed at least 2 weeks from the initial imaging or at the next available date in the event of clinical deterioration to obtain differential diagnosis (e.g. rupture of abscess and concomitant pathology). Parenteral antibiotics were converted to oral formulations based on culture sensitivity results, clinical progress and downtrend of inflammatory markers. Duration of antibiotic therapy was guided by clinical judgement, biochemical and radiological progress; antibiotics were discontinued on clinical resolution and/or near radiological resolution (absence or almost complete reduction in size of PLA on imaging). Follow-up elective interval cholecystectomy was offered for patients with gallstones. Colonic evaluation with colonoscopy was also advised.

### Statistical Analysis

Mean imputation was performed for missing data values where < 10% was missing. The Shapiro-Wilk test of normality was performed for all continuous variables and revealed a nonparametric distribution for all continuous variables except for haemoglobin (*P* = 0.514) and albumin (*P* = 0.337). Categorical variables were expressed as number (%) and were analysed by Pearson’s chi-squared test or Fisher’s exact test if expected cell count < 5. Median (interquartile range [IQR]) values were used for all continuous variables as the majority of the variables followed nonparametric distributions and were analysed by the Mann-Whitney U test. PSM was performed using logistic regression. PSM was performed at a ratio of 1:1 using a caliper width of 0.1 of the standard deviation of the logit of the propensity score (28). Patients were adjusted for 13 variables: 10 variables (ASA score ≥ 3, presence of hypertension, hyperlipidaemia [use of statins], diabetes mellitus, raised bilirubin > 31 μmol/L, creatinine > 176 μmol/L, albumin < 25 g/L, alanine aminotransferase (ALT), presence of multiple abscesses and gas) were demonstrated to prognosticate outcomes in PLA and/or were significantly different between the two groups (8, 11, 29–31); three variables (presence of renal impairment, ischaemic heart disease and haemoglobin) were significantly different between the two groups. We did not include gallstone etiology in PSM as this was unlikely to influence short-term outcomes, compared to the presence of cholecystitis, for which we did not collect data on. Logistic regression was used for multivariate analysis to assess the impact of age on outcomes using the same variables used for PSM in both the unmatched and matched cohorts. Standardised mean difference (SMD) and, Hansen and Bowers test were used to assess covariable and global imbalance, respectively (32). Statistical significance was defined as *P* < 0.05. All statistical analyses were performed with SPSS version 25.0 (SPSS Inc., Chicago, III., United States) and *R* software (R-3.3.3).

## Results

### Baseline Demographics and Clinical Profile

A total of 213 patients with PLA (elderly patients = 90 [42.3%] and non-elderly patients = 123 [57.7%]) were included in this study period. Overall median age is 62 (IQR = 53–74) years old with male predominance (*n* = 131/213, 61.5%). The most common comorbidities were hypertension (*n* = 100/213 [47.0%]), hyperlipidemia (*n* = 88/213 [41.3%]) and diabetes mellitus (*n* = 74/213 [34.7%]). There were 96/213 (45.1%) and 83/122 (68.0%) positive blood and pus cultures, respectively. The median size of abscess was 5.4 (IQR = 3.9–7.4) cm and gas-forming PLA (GFPLA) was present in 41 patients (19.2%). In the unmatched cohort, baseline demographics were significantly different, including higher ASA score, presence of co-morbidities (diabetes mellitus, hypertension, hyperlipidemia, renal impairment and ischaemic heart disease) and worse biochemistry markers (haemoglobin, ALT and creatinine) in the elderly group (Table 1). There were 13 (6.1%) patients with PLA ≥ 10 cm.

**Figure 1:**
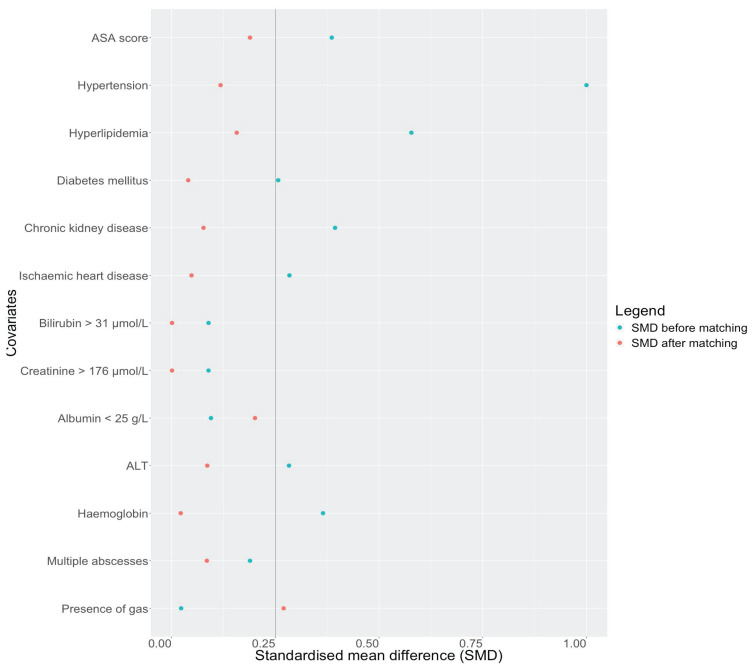
Plot showing the standardised mean difference (SMD) in covariates before PSM (blue) and after PSM (red). SMD of < 0.25 indicates adequate balance; ASA = American Association of Anesthesiologists; ALT = alanine aminotransferase; PSM = Propensity score matching

PSM was performed in a 1:1 ratio resulting in 102 patients (elderly patients = 51 and non-elderly patients = 51). Before PSM, there were eight variables with SMD > 0.25, while there was one variable with SMD > 0.25 after PSM (Figure 1). Hansen and Bowers test for global significance did not show any significant difference in the matched cohort (after PSM: χ^2^: 6.06, *P* = 0.944; before PSM: χ^2^: 55.7, *P* < 0.001). This suggests an improved balance after PSM. Most of the baseline demographics were comparable in the matched cohort after PSM, except for median aspartate aminotransferase (AST) (elderly patients = 68 IU/L versus non-elderly patients = 48 IU/L, *P* = 0.046). Baseline demographics and clinical profiles of both unmatched and matched cohorts are summarised in Table 1.

### Clinical Outcomes

Table 2 summarises the outcomes between elderly patients and non-elderly patients in both our unmatched and matched cohorts. The median LOS was 14 (IQR = 8–21) days. There were 28 patients (13.1%) with in-hospital mortality. In our unmatched cohort, LOS was significantly longer in elderly patients (elderly patients: 16 [IQR = 10–24.5] days, non-elderly patients: 11 [IQR = 8–19] days, *P* < 0.001). This was similarly noted in our matched cohort (elderly patients: 17 [IQR = 13–27] days, non-elderly patients: 11 [IQR = 7–19] days, *P* = 0.001). Duration of parenteral and total antibiotic therapy, need for PD and 30-day re-admission were comparable between elderly patients and non-elderly patients in the unmatched and matched cohort. In-hospital mortality was significantly higher in elderly patients in the unmatched cohort (elderly patients: 19 [21.1%], non-elderly patients: 9 [7.3%], OR = 3.39 [95% CI: 1.45, 7.90], *P* = 0.003). Following PSM, there was a trend towards higher in-hospital mortality in elderly patients though statistical significance was not met (elderly patients: 8 [15.7%], non-elderly patients: 5 [9.8%], OR = 2.88 [95% CI: 0.53, 15.51], *P* = 0.219).

### Subgroup Analysis of Patients Who Underwent Percutaneous Drainage

A total of 122 (57.3%) and 66 (64.7%) patients underwent PD in the unmatched and matched cohorts, respectively. In the unmatched cohort, LOS was significantly longer in the elderly patients compared to non-elderly patients (elderly patients: 18 [IQR = 13–26] days versus non-elderly patients: 13 [IQR = 8–20] days, *P* = 0.012) and more elderly patients had LOS > 14 days (OR = 4.26 [95% CI: 1.61, 11.30], *P* = 0.004). LOS was similarly longer in elderly patients in the matched cohort but was not statistically significant (elderly patients: 17 [IQR = 13–26.8] days versus non-elderly patients: 13.5 [IQR 8–21.3] days, *P* = 0.063). More elderly patients similarly had LOS > 14 days (OR = 10.12 [95% CI: 1.94, 52.93], *P* = 0.006). In-hospital mortality was comparable between elderly and non-elderly patients in both the unmatched (elderly patients: *n* = 11/55 [20.0%], non-elderly patients: *n* = 3/67 [4.5%], OR = 2.70 [95% CI: 0.32, 23.10], *P* = 0.364) and matched cohorts (elderly patients: *n* = 4/36 [11.1%], non-elderly patients: *n* = 3/30 [10.0%], OR = 0.20 [95% CI: 0.01, 7.75], *P* = 0.394).

## Discussion

This single-centre PSM study demonstrated that age ≥ 65 years old is associated with longer LOS and a non-statistically significantly higher mortality. The elderly population is expected to increase with an increase in global life expectancy and advancement in healthcare. While old age is associated with more comorbidities, there are elderly patients with little or no comorbidities. Both old age and the presence of comorbidities result in diminished reserves and functional decline; it is, therefore, essential to evaluate whether age alone affects outcomes.

The association of old age with poorer outcomes in PLA had been previously shown in the literature. Chen et al. (18) retrospectively reviewed 339 patients (age ≥ 65 years old: *n* = 118 [34.8%], age < 65 years old: *n* = 221 [65.2%]) and demonstrated that age ≥ 65 years old was associated with longer LOS (age ≥ 65 years old: 25.5 ± 22.7 days, age < 65 years old: 19.5 ± 10.7 days, *P* = 0.008), longer duration of parenteral antibiotics (age ≥ 65 years old: 21.7 ± 20.0 days, age < 65 years old: 18.1 ± 10.8 days, *P* = 0.033), with comparable mortality (age ≥ 65 years old: *n* = 16 [13.6%], age < 65 years old: *n* = 19 [8.6%], *P* = 0.153). However, it is prudent to note that their study only reported comorbidities of biliary stone disorder, malignancy and alcoholism. Their analysis (18) did not include common but clinically important comorbidities such as diabetes mellitus, hypertension and ischaemic heart disease.

Another study by Law and Li (19) on 319 patients (age ≥ 65 years old: *n* = 168 [52.7%], age < 65 years old: *n* = 151 [47.3%]) showed that age ≥ 65 years old was associated with higher in-hospital mortality rate (age ≥ 65 years old: *n* = 37 [22.0%], age < 65 years old: *n* = 14 [9.3%], *P* < 0.01) and higher PLA recurrence (age ≥ 65 years old: *n* = 13 [7.7%] versus age < 65 years old: *n* = 4 [2.6%], *P* = 0.02). In patients with age ≥ 65 years old, there was higher incidence of hypertension (39.3% versus 17.9%, *P* < 0.01), ischaemic heart disease (13.1% versus 4.0%, *P* < 0.01) and stroke (16.1% versus 4.0%, *P* < 0.01).

Our study similarly showed higher incidence of hypertension and ischaemic heart disease incidence in age ≥ 65 years old, which is unsurprising. We did PSM due to the presence of multiple confounding factors, including presence of comorbidities. We found longer LOS in elderly patients in both our unmatched and matched cohorts. In our unmatched cohort, in-hospital mortality was significantly higher in elderly patients (elderly patients: 19 [21.1%], non-elderly patients: 9 [7.3%], OR = 3.39 [95% CI: 1.45, 7.90], *P* = 0.003). However, we failed to obtain statistical significance following PSM (elderly patients: 8 [15.7%], non-elderly patients: 5 [9.8%], OR = 2.88 [95% CI: 0.53, 15.51], *P* = 0.219). Nevertheless, mortality of 15.7% is considered clinically significantly higher than 9.8% (absolute difference of 5.9%) and this deserves to be mentioned. This result is similar to the study by Chen et al. (18), where mortality was 13.6% in age ≥ 65 years old and 8.6% in age < 65 years old (absolute difference of 5.0%), though not statistically significant (*P* = 0.153). Failure to reach statistical significance may be due to a small sample size (33).

The overall mortality of 13.1% reported by our study is acceptable and is at the lower spectrum of internationally reported mortality of 10%–40% (34). Our institution employs a ‘liver abscess care bundle’ in the management of PLA, integrating surgical, microbiology, interventional radiology and nursing teams for multidisciplinary management (5). The surgical team provides an overall management, the microbiology team provides prompt blood culture advisory for culture-directed antibiotics and transition to outpatient antibiotic therapy if required, the interventional radiology team provides round-the-clock service for PD and tube reviews, and the nursing team provides good drain care and discharge advice. The implementation of this care bundle may explain the relatively low mortality in our series.

The incidence of GFPLA is reported to be 7%–24% and is traditionally associated with higher mortality ranging from 25.7% to 37.1%, compared to non-GFPLA with mortality 4.1%–14.4% (10, 35–40). The overall incidence of GFPLA in our study was 41/213 (19.2%) which is comparable to internationally reported incidence. Given its association with septic shock and mortality, ‘presence of gas’ was one of the variables included in our PSM model. Unfortunately, the SMD increased from 0.023 before PSM to 0.270 after PSM, suggesting a lack of balance. This is a limitation in order to obtain good matching for the other variables. Chan et al. (11) compared outcomes of GFPLA versus non-GFPLA in a matched cohort and showed no significant differences in LOS (GFPLA: 14 [IQR = 8–19] days versus non-GFPLA: 15 [IQR = 8–22] days, *P* = 0.299), duration of antibiotic use (GFPLA: 39 [IQR = 26–49] days versus non-GFPLA: 37 [IQR = 28–49] days, *P* = 0.634), need for PD (GFPLA: *n* = 26/36 [72.2%], non-GFPLA: *n* = 47/72 [65.3%], *P* = 0.467) and in-hospital mortality (GFPLA: *n* = 4/36 [11.1%] versus non-GFPLA: *n* = 7/76 [9.7%], *P* = 0.822). The presence of gas alone may not be predictive of poor outcomes. In addition, though PSM was unable to obtain adequate balance in our matched cohort, we also subsequently performed a multivariate analysis in the matched cohort and included GFPLA as a covariate to address its potential confounding effect on outcomes.

Size of abscess is also a predictor of outcomes, with literature quoting various size cut-offs ranging 2 cm–5 cm to determine the need for PD (5, 41, 42). The theory behind this is through the calculation of the volume of abscess and the mathematical concept of a sphere: the size of PLA of diameters 3 cm, 4 cm and 5 cm correspond to estimated volumes of 14 cc, 33.5 cc and 65 cc, respectively, with volume doubling significantly as PLA size increase from 4 cm to 5 cm (5). Hence, our institution uses a cut-off of 4 cm for PD. We did a subgroup analysis of patients who required PD (i.e. based on size cut-off, haemodynamic instability or failure of conservative treatment); we demonstrated that age ≥ 65 years old is associated with LOS > 14 days in both the unmatched (OR = 4.26 [IQR = 1.61–11.30], *P* = 0.004) and matched cohorts (OR = 10.12 [IQR = 1.94–52.93], *P* = 0.006) (Table 3). Interestingly, multivariate analysis of patients who required PD did not demonstrate any statistical significance in outcomes between elderly and non-elderly patients in the unmatched and matched cohorts. Our matched cohort further showed comparable incidence of in-hospital mortality (elderly patients: 11.1% versus non-elderly patients: 10.0%, OR = 0.20 [95% CI: 0.01, 7.75], *P* = 0.394). It is possible that PD allows for early source control and improves outcomes. This has been shown by Lo et al. (8) in their multivariate analysis of 311 patients (mean age for patients with resolution of PLA: 58.4 ± 15.4 years old versus failure of therapy: 66.1 ± 14.7 years old), who required PD, of which age was not a predictor of failure of PD. However, we caution to interpret the results as such. In the unmatched cohort, we obtained a mean difference of 15.5% in incidence of in-hospital mortality (overall cohort: elderly patients *n* = 11/55 (20.0%); non-elderly patients *n* = 3/67 (4.5%), OR = 2.70 [95% CI: 0.32, 23.10], *P* = 0.364). This mean difference is similar to that of our overall cohort (elderly patients *n* = 19/90 [21.1%], non-elderly *n* = 9/123 [7.3%], mean difference = 13.8%). The incidence of 20% mortality versus 4.5% is clinically significant. However, following multivariate analysis, there was a lack of statistical significance in the subgroup of patients who underwent PD, compared to the overall cohort. This may be due to the small sample size, along with the large number of variables used in multivariate analysis. Hence, we take caution to interpret that in-hospital mortality is comparable between elderly and non-elderly who underwent PD.

Another issue of discussion is the microbiology of PLA and increasing drug resistance globally which may affect outcomes. Our study showed that *Klebsiella pneumoniae* was the most common organism, followed by *Escherichia coli*; this was similar between elderly and non-elderly in both the unmatched and matched cohorts. Locally, we adopt the use of amoxicillin-clavulanic acid and stat dose of gentamicin for empiric coverage in patients presenting with hepatobiliary sepsis as per our local antibiotogram. While we did not collect data on antimicrobial sensitivity, this data has been previously reported in our local context. For instance, Hsu et al. (43) in 2010 evaluated the antimicrobial resistance in four local hospitals from 2006 to 2008 and showed that *Klebsiella pneumoniae* and *Escherichia coli* has 32.3% and 20.0% resistance to ceftriaxone, respectively. Extended-spectrum beta-lactamase (ESBL) producing *Klebsiella pneumoniae* and *Escherichia coli* has been demonstrated to be 30.1% and 19.6%, respectively, by Tan et al. (44) in 2008. This trend of antimicrobial resistance has been stable over the last decade has been reported by Chua et al. (45) in 2019. While it is possible that antimicrobial sensitivity may be a confounding factor, this is less likely due to regular audits by our department on the antibiotic stewardship programme which regulates the use on the type of empiric antibiotics.

Our study has its strengths. To our knowledge, this is the first PSM study evaluating the impact of age (65 years old as cut-off) on outcomes in PLA. While age is commonly associated with comorbidity, there are elderly patients with little or no comorbidity and are not limited in their functional status (represented by ASA score). In addition, the use of PSM allows minimisation of selection bias and ranks superior in hierarchy compared to existing non-matched observational comparative studies (46). However, this study has its limitations. After PSM, there was a variable (presence of GFPLA) that had SMD > 0.25 (which was < 0.25 before PSM). We attempted to improve the matching by decreasing caliper width to 0.1 and reducing variables included in the PSM analysis, but this was the best outcome. Hence, we addressed this limitation by further addressing the potential confounding effect using multivariate logistic regression to assess outcomes. In addition, subgroup analysis of patients with PD resulted in a small sample size which we caution interpretation of the lack of statistical significance in mortality rates, as incidence appears to be clinically significant. Global incidence of PLA is low, though relatively more common in Asian population. Existing studies which reported on PLA had sample sizes ranging from 80 to 352. A sample size of 398 (199 patients in each arm) is required to find a 10% difference in mortality with 80% power and two-sided alpha of 5%.

The lack of statistical significance in in-hospital mortality following PSM in our study may be due to small sample size. We did not collect data on in-hospital complications such as incidence of pneumonia, cardiovascular events and renal impairment, which may prolong LOS. Elderly patients have lower physiological reserves which may result in longer LOS in the event of complications. We also did not collect data on antibiotic sensitivity, failure of antibiotic therapy, long-term recurrence rate and patients who required surgical drainage. Data on antibiotic sensitivity patterns and presence of ESBL organisms has been previously reported by local authors as described in our discussion above (43–45). Lastly, we were only possible to retrieve data from 2007–2011 given institutional policies. The inclusion of more recent data may show improved outcomes with continued advancements in medical care and interventional radiology techniques.

## Conclusion

Age ≥ 65 years old is associated with an increased LOS. While increased in-hospital mortality was statistically significant in our unmatched cohort, this was comparable in the matched cohort. Whether this is due to sampling size limitation is yet to be determined, as the proportion of elderly with in-hospital mortality remains clinically significant and higher than non-elderly patients after matching. Therefore, age should be considered for severity stratification for PLA. However, further large sample studies should be conducted to validate our findings.

## Figures and Tables

**Table 1:** Demographics and clinical profile of elderly versus non-elderly patients with PLA

	Overall cohort, *n* = 213				PSM cohort, *n* = 102			

	Elderly *n* = 90 (%)	Non-elderly *n* = 123 (%)	*P-*value	SMD	Elderly *n* = 51 (%)	Non-elderly *n* = 51 (%)	*P*-value	SMD
Age, median (IQR)	75 (68.8–80)	54 (42–60)	**< 0.001**	-	74 (68–80)	57 (50–62)	**< 0.001**	-
ASA, median (IQR)	2 (2–2)	1 (1–2)	**< 0.001**	-	2 (1–2)	2 (1–2)	0.093	-
≥ 3, yes*	14 (15.6)	5 (4.1)	**0.004**	0.386	7 (13.7)	4 (7.8)	0.338	0.189
Gender, male	50 (55.6)	81 (65.9)	0.127	-	31 (60.8)	31 (60.8)	1.000	-
Comorbidities								
Diabetes mellitus*	37 (41.1)	37 (30.1)	**0.095**	0.257	19 (37.3)	21 (41.2)	0.685	0.040
Hypertension*	66 (73.3)	34 (27.6)	**< 0.001**	1.048	27 (52.9)	30 (58.8)	0.550	0.118
Renal impairment*	13 (14.4)	4 (3.3)	**0.003**	0.394	3 (5.9)	4 (7.8)	0.695	0.077
Chronic obstructive pulmonary disease	8 (8.9)	5 (4.1)	0.146	-	4 (7.8)	1 (2.0)	0.169	-
Ischaemic heart disease*	21 (23.3)	15 (12.2)	**0.032**	0.284	10 (19.6)	11 (21.6)	0.807	0.048
Hyperlipidaemia*	51 (56.7)	37 (30.1)	**< 0.001**	0.578	24 (47.1)	21 (41.2)	0.550	0.157
Thyroid disease	1 (1.1)	3 (2.4)	0.481	-	0 (0)	2 (3.9)	0.153	-
Clinical presentation								-
Fever	72 (80.0)	103 (83.7)	0.481	-	43 (84.3)	43 (84.3)	1.000	-
Jaundice	3 (3.3)	7 (5.7)	0.422	-	1 (2.0)	6 (11.8)	0.112	-
Abdominal pain	41 (45.6)	70 (56.9)	0.101	-	22 (43.1)	26 (51.0)	0.427	-
Septic shock	6 (6.7)	10 (8.1)	0.689	-	3 (5.9)	6 (11.8)	0.487	-
Cause, gallstone	48 (53.3)	40 (32.5)	**0.002**	-	28 (54.9)	24 (47.1)	0.428	-
Haematological investigations								
Haemoglobin (g/dL)*	12.0 (10.7–12.9)	12.5 (11.4–13.8)	**0.011**	0.365	12.3 (10.9–13.2)	11.7 (10.3–13.6)	0.825	0.022
White blood cells (10^9^/L)	13.5 (9.5–17.4)	14.1 (9.9–17.6)	0.778	-	13.6 (8.9–16.7)	13.2 (9.6–15.9)	0.512	-
Platelets (10^9^/L)	213 (127–329)	215 (152–343)	0.617	-	225 (125–338)	229 (168–312)	0.651	-
International normalised ratio	1.22 (1.12–1.34)	1.22 (1.11–1.35)	0.800	-	1.20 (1.12–1.38)	1.21 (1.14–1.28)	0.431	-
Creatinine (μmol/L)	113 (90–155)	93 (75–130)	**0.005**	-	102 (77–144)	93 (75–130)	0.186	-
> 176, yes*	14 (15.6)	15 (12.2)	0.480	0.089	7 (13.7)	7 (13.7)	1.000	< 0.001
Total bilirubin (μmol/L)	26 (16–39.5)	25 (15–38)	0.776	-	25 (16–39)	22 (14–37)	0.558	-
> 31, yes*	36 (40.0)	43 (35.0)	0.452	0.089	18 (35.3)	18 (35.3)	1.000	< 0.001
ALT (IU/L) *	58 (30–88)	74 (37–113)	**0.031**	0.283	59 (35–94)	47 (30–97)	0.899	0.086
AST (IU/L)	67 (38–100)	55 (34–95)	0.254	-	68 (38–101)	48 (27–85)	**0.046**	-
ALP (IU/L)	143 (89–213)	139 (96–199)	0.727	-	146 (98–260)	131 (88–202)	0.314	-
GGT (IU/L)	105 (51–222)	126 (55–180)	0.929	-	108 (43–224)	108 (55–192)	0.901	-
Albumin (g/L)	27 (23.8–31)	26 (22–31)	0.702	-	25 (16–39)	26 (21–30)	0.812	-
< 25, yes*	27 (30.0)	44 (35.8)	0.377	0.095	22 (43.1)	18 (35.3)	0.417	0.201
Blood culture (positive)	46 (51.1)	50 (40.7)	0.130	-	23 (45.1)	20 (39.2)	0.547	-
*Klebsiella pneumoniae*	31 (67.4)	36 (72.0)			16 (69.6)	13 (65.0)		
*Escherichia coli*	5 (10.9)	3 (6.0)			2 (8.7)	1 (5.0)		
*Pseudomonas aeruginosa*	0 (0)	2 (4.0)			0 (0)	1 (5.0)		
Others	10 (21.7)	9 (18.0)			5 (21.7)	5 (25.0)		
Pus culture (positive)#	38 (69.1)	45 (67.2)	0.405	-	26 (77.8)	22 (73.3)	0.427	-
*Klebsiella pneumoniae*	32 (84.2)	36 (80.0)			22 (84.6)	16 (72.7)		
*Escherichia coli*	2 (5.3)	0 (0)			1 (3.9)	0 (0)		
*Clostridium perfringes*	1 (2.6)	1 (2.2)			1 (3.9)	0 (0)		
Others	3 (7.9)	8 (17.8)			2 (7.7)	6 (27.3)		
Radiological investigations								
Number of abscess, multiple*	35 (38.9)	36 (29.3)	0.141	0.189	16 (31.4)	16 (31.4)	1.000	0.085
Size of largest abscess (cm)	5.4 (4.0–7.5)	5.6 (3.8–7.3)	0.854	-	5.4 (4.0–7.0)	5.9 (4.7–7.5)	0.393	-
Presence of gas*	18 (20.0)	23 (18.7)	0.812	0.023	16 (31.4)	10 (19.6)	0.173	0.270

Notes:

* PSM was performed for these variables due to potential and/or significant effects on clinical outcomes;

# The incidence of pus culture is expressed as a percentage of patients who had PD; All categorical variables are reported in *n* (%), and all continuous variables are reported in median (IQR);

ALP = alkaline phosphatase; Values in bold indicates p<0.100, which were variables considered to be used for propensity score matching; ALT = alanine aminotransferase; AST = aspartate aminotransferase; GGT = gamma-glutamyl transferase; PSM = propensity score matching; SMD = standardised mean difference; IQR = interquartile range

**Table 2:** Outcomes of elderly versus non-elderly patients with PLA

	Overall cohort, *n* = 213				PSM cohort, *n* = 102			

	Elderly *n* = 90 (%)	Non-elderly *n* = 123 (%)	OR (95% CI)*	*P-*value*	Elderly *n* = 51 (%)	Non-elderly *n* = 51 (%)	OR (95% CI)*	*P-*value*
Length of stay (days), median (IQR)	16 (10–24.5)	11 (8–19)		< 0.001	17 (13–27)	11 (7–19)		0.001
> 7 days	83 (92.2)	93 (75.6)	3.83 (1.60, 9.17)	**0.002**	47 (92.2)	36 (70.6)	5.62 (1.28, 24.62)	**0.022**
> 14 days	57 (63.3)	44 (35.8)	3.10 (1.76, 5.46)	**< 0.001**	36 (70.6)	18 (35.3)	7.01 (2.44, 20.44)	**< 0.001**
Duration of antibiotics (days), median (IQR)								
Parenteral	13 (7–17.3)	11.5 (7–14.6)	-	0.254	12 (7–16)	12 (7–14.6)		0.740
Total (parenteral and oral)	39 (28–49.3)	39 (26.6–49)	-	0.272	39 (29–48)	38 (28–49)		0.623
PD, yes	55 (61.1)	67 (54.5)	1.31 (0.76, 2.28)	0.333	36 (70.6)	30 (58.8)	1.77 (0.69, 4.57)	0.239
Duration of drain (days), median (IQR)	4.5 (1–7)	5 (3–7)	-	0.608	4 (1–7)	5 (2.8–8)		0.336
30-day re-admission	13 (14.4)	16 (13.0)	1.13 (0.51, 2.48)	0.763	8 (15.7)	7 (13.7)	1.56 (0.34, 6.86)	0.559
In-hospital mortality	19 (21.1)	9 (7.3)	3.39 (1.45, 7.90)	**0.003**	8 (15.7)	5 (9.8)	2.88 (0.53, 15.51)	0.219

Notes:

* Binomial variables were analysed using multivariate logistic regression using the variables used for PSM; All categorical variables are reported in *n* (%) and all continuous variables are reported in median (IQR);

CI = confidence interval; IQR = interquartile range; OR = odds ratio; PLA = pyogenic liver abscess; PSM = propensity score matching

**Table 3:** Subgroup analysis of outcomes with patients who underwent percutaneous drainage

	Overall cohort, n = 122				PSM cohort, n = 66			
	Elderly n = 55 (%)	Non-elderly n = 67 (%)	OR (95% CI)*	P-value*	Elderly n = 36 (%)	Non-elderly n = 30 (%)	OR (95% CI)*	P-value*
Length of stay (days), median (IQR)	18 (13–26)	13 (8–20)	**-**	**0.012**	17 (13–26.8)	13.5 (8–21.3)	-	0.063
> 7 days	52 (94.5)	59 (88.1)	4.15 (0.77, 22.41)	0.099	34 (94.4)	25 (83.3)	16.87 (0.41, 702.62)	0.138
> 14 days	38 (69.1)	27 (40.3)	4.26 (1.61, 11.30)	**0.004**	25 (69.4)	12 (40.0)	10.12 (1.94, 52.93)	**0.006**
Duration of antibiotics (days), median (IQR)								
Parenteral	14 (9–19)	11.5 (8–14.6)	-	0.274	12 (8.3–16.8)	13 (9.5–14.6)	-	0.959
Total (parenteral and oral)	39 (29–50)	38.7 (28–51)	-	0.652	40 (35–49.8)	38.4 (29.4–53.9)	-	0.709
Duration of drain (days), median (IQR)	4.5 (1–7)	5 (3–7)	-	0.120	4 (1–7)	5 (2.8–8)	-	0.336
30-day readmission	8 (14.5)	7 (10.4)	0.43 (0.09, 2.09)	0.298	4 (11.1)	4 (13.3)	0.45 (0.03, 6.37)	0.556
In-hospital mortality	11 (20.0)	3 (4.5)	2.70 (0.32, 23.10)	0.364	4 (11.1)	3 (10.0)	0.20 (0.01, 7.75)	0.394

Notes:

* Binomial variables were analysed using multivariate logistic regression using the variables used for PSM; All categorical variables are reported in *n* (%), and all continuous variables are reported in median (IQR);

CI = confidence interval; IQR = interquartile range; OR = odds ratio; PLA = pyogenic liver abscess; PSM = propensity score-matched

## References

[1] Altemeier W, Culbertson W, Fullen W, Shook CD (1973). Intra-abdominal abscesses. Am J Surg.

[2] Tian LT, Yao K, Zhang XY, Zhang ZD, Liang YJ, Yin DL (2012). Liver abscesses in adult patients with and without diabetes mellitus: an analysis of the clinical characteristics, features of the causative pathogens, outcomes and predictors of fatality: a report based on a large population, retrospective study in China. Clin Microb Infect.

[3] Lardière-Deguelte S, Ragot E, Amroun K, Piardi T, Dokmak S, Bruno O (2015). Hepatic abscess: diagnosis and management. J Visc Surg.

[4] Branum GD, Tyson GS, Branum MA, Meyers WC (1990). Hepatic abscess: changes in etiology, diagnosis, and management. Annals Surg.

[5] Shelat VG, Chia CLK, Yeo CSW, Qiao W, Woon W, Junnarkar SP (2015). Pyogenic liver abscess: does *Escherichia coli* cause more adverse outcomes than *Klebsiella pneumoniae*?. World J Surg.

[6] Ahmed S, Chia CLK, Junnarkar SP, Woon W, Shelat VG (2016). Percutaneous drainage for giant pyogenic liver abscess—is it safe and sufficient?. Am J Surg.

[7] Shelat VG, Wang Q, Chia CL, Wang Z, Low JK, Woon WW (2016). Patients with culture negative pyogenic liver abscess have the same outcomes compared to those with Klebsiella pneumoniae pyogenic liver abscess. Hepatob Pancreat Dis Int.

[8] Lo JZW, Leow JJJ, Ng PLF, Lee HQ, Mohd Noor NA, Low JK (2015). Predictors of therapy failure in a series of 741 adult pyogenic liver abscesses. J Hepatobiliary Pancreat Sci.

[9] Danson Y, Yuan TM, Shelat VG, Sartelli M, Bassetti M, Martin-Loeches I (2018). Pyogenic liver abscess. Abdominal sepsis: a multidisciplinary approach.

[10] Thng CB, Tan YP, Shelat VG (2018). Gas-forming pyogenic liver abscess: a world review. Ann Hepatobiliary Pancreat Sci.

[11] Chan KS, Thng CB, Chan Y-H, Shelat VG (2020). Outcomes of gas-forming pyogenic liver abscess are comparable to non-gas-forming pyogenic liver abscess in the era of multi-modal care: a propensity score matched study. Surg Infect.

[12] Lim WS, van der Eerden MM, Laing R, Boersma WG, Karalus N, Town GI (2003). Defining community acquired pneumonia severity on presentation to hospital: an international derivation and validation study. Thorax.

[13] Gharbi M, Drysdale JH, Lishman H, Goudie R, Molokhia M, Johnson AP (2019). Antibiotic management of urinary tract infection in elderly patients in primary care and its association with bloodstream infections and all cause mortality: population based cohort study. BMJ.

[14] Kiriyama S, Kozaka K, Takada T, Strasberg SM, Pitt HA, Gabata T (2018). Tokyo Guidelines 2018: diagnostic criteria and severity grading of acute cholangitis (with videos). J Hepatobiliary Pancreat Surg.

[15] Ong Y, Shelat VG (2021). Ranson score to stratify severity in acute pancreatitis remains valid—old is gold. Expert Rev Gastroenterol Hepatol.

[16] Blamey SL, Imrie CW, O’Neill J, Gilmour WH, Carter DC (1984). Prognostic factors in acute pancreatitis. Gut.

[17] Mohan R, Huey CWT, Junnarkar S, Low JK, Shelat VG (2020). Prehabilitation in elderly patients scheduled for liver resection and protocol for recovery of surgery in elderly. Hepatoma Res.

[18] Chen S-C, Lee Y-T, Yen C-H, Lai K-C, Jeng L-B, Lin D-B (2009). Pyogenic liver abscess in the elderly: clinical features, outcomes and prognostic factors. Age Ageing.

[19] Law S-T, Li KK (2013). Older age as a poor prognostic sign in patients with pyogenic liver abscess. Int J Infect Dis.

[20] Chan KS, Mohan R, Low JK, Junnarkar SP, Huey CWT, Shelat VG (2021). Elderly patients (≥ 80 years) with acute calculous cholangitis have similar outcomes as non-elderly patients (< 80 years): propensity score-matched analysis. World J Hepatol.

[21] Ouchi Y, Rakugi H, Arai H, Akishita M, Ito H, Toba K (2017). Redefining the elderly as aged 75 years and older: proposal from the Joint Committee of Japan Gerontological Society and the Japan Geriatrics Society. Geriatr Gerontol Int.

[22] Sulpice L, Rayar M, Campillo B, Pery C, Guillaud A, Meunier B (2014). Advanced age remains an achilles heel for liver resections. World J Surg.

[23] Tzeng CW, Cooper AB, Vauthey JN, Curley SA, Aloia TA (2014). Predictors of morbidity and mortality after hepatectomy in elderly patients: analysis of 7621 NSQIP patients. HPB (Oxford).

[24] Suzuki T, Kwon J (2006). Cross sectional and longitudinal study on the health status among the Japanese elderly from prospective cohort study. Jpn J Health and Welfare (Kousei no Shihyou).

[25] Von Elm E, Altman DG, Egger M, Pocock SJ, Gøtzsche PC, Vandenbroucke JP (2007). The Strengthening the Reporting of Observational Studies in Epidemiology (STROBE) statement: guidelines for reporting observational studies. Ann Intern Med.

[26] Dellinger RP, Levy MM, Rhodes A, Annane D, Gerlach H, Opal SM (2013). Surviving sepsis campaign: international guidelines for management of severe sepsis and septic shock, 2012. J Intensive Care Med.

[27] Cai Y-L, Xiong X-Z, Lu J, Cheng Y, Yang C, Lin Y-X (2015). Percutaneous needle aspiration versus catheter drainage in the management of liver abscess: a systematic review and meta-analysis. HPB.

[28] Austin PC (2011). Optimal caliper widths for propensity-score matching when estimating differences in means and differences in proportions in observational studies. Pharm Stat.

[29] Lee CH, Jo HG, Cho EY, Song JS, Jung GM, Cho YK (2021). Maximal diameter of liver abscess independently predicts prolonged hospitalization and poor prognosis in patients with pyogenic liver abscess. BMC Infect Dis.

[30] Chou FF, Sheen-Chen SM, Chen YS, Chen MC, Chen FC, Tai DI (1994). Prognostic factors for pyogenic abscess of the liver. J Am Coll Surg.

[31] Liao KF, Cheng KC, Lin CL, Lai SW (2017). Statin use correlates with reduced risk of pyogenic liver abscess: a population-based case-control study. Basic Clin Pharmacol Toxicol.

[32] Austin PC (2009). Balance diagnostics for comparing the distribution of baseline covariates between treatment groups in propensity-score matched samples. Stat Med.

[33] Ranganathan P, Pramesh CS, Buyse M (2015). Common pitfalls in statistical analysis: clinical versus statistical significance. Perspect Clin Res.

[34] Huang C-J, Pitt HA, Lipsett PA, Osterman FAJ, Lillemoe KD, Cameron JL (1996). Pyogenic hepatic abscess: changing trends over 42 years. Annals Surg.

[35] Lee H-L, Lee H-C, Guo H-R, Ko W-C, Chen K-W (2004). Clinical significance and mechanism of gas formation of pyogenic liver abscess due to *Klebsiella pneumoniae*. J Clin Microbiol.

[36] Yang C-C, Chen C-Y, Lin X-Z, Chang T-T, Shin J-S, Lin CY (1993). Pyogenic liver abscess in Taiwan: emphasis on gas-forming liver abscess in diabetics. Am J Gastroenterol.

[37] Chou F-F, Sheen-Chen S-M, Chen Y-S, Lee T-Y (1995). The comparison of clinical course and results of treatment between gas-forming and non-gas-forming pyogenic liver abscess. Arch Surg.

[38] Lee T-Y, Wan Y-L, Tsai C-C (1994). Gas-containing liver abscess: radiological findings and clinical significance. Abdom Imaging.

[39] Chen S-C, Huang C-C, Tsai S-J, Yen C-H, Lin D-B, Wang P-H (2009). Severity of disease as main predictor for mortality in patients with pyogenic liver abscess. Am J Surg.

[40] Foo N-P, Chen K-T, Lin H-J, Guo H-R (2010). Characteristics of pyogenic liver abscess patients with and without diabetes mellitus. Am J Gastroenterol.

[41] Sayek I, Onat D, Holzheimer RG, Mannick JA (2001). Pyogenic and amebic liver abscess. Surgical treatment: evidence-based and problem-oriented.

[42] Hope WW, Vrochides DV, Newcomb WL, Mayo-Smith WW, Iannitti DA (2008). Optimal treatment of hepatic abscess. Am Surg.

[43] Hsu L-Y, Tan T-Y, Tam VH, Kwa A, Fisher DA, Koh T-H (2010). Surveillance and correlation of antibiotic prescription and resistance of Gram-negative bacteria in Singaporean hospitals. Antimicrob Agents Chemother.

[44] Tan TY, Hsu LY, Koh TH, Ng LS, Tee NW, Krishnan P (2008). Antibiotic resistance in gram-negative bacilli: a Singapore perspective. Ann Acad Med Singap.

[45] Chua AQ, Kwa AL, Tan TY, Legido-Quigley H, Hsu LY (2019). Ten-year narrative review on antimicrobial resistance in Singapore. Singapore Med J.

[46] Rosenbaum PR, Rubin DB (1983). The central role of the propensity score in observational studies for causal effects. Biometrika.

